# A Reverse Stroop Task with Mouse Tracking

**DOI:** 10.3389/fpsyg.2016.00670

**Published:** 2016-05-06

**Authors:** Naohide Yamamoto, Sara Incera, Conor T. McLennan

**Affiliations:** ^1^School of Psychology and Counselling and Institute of Health and Biomedical Innovation, Queensland University of Technology, Kelvin GroveQLD, Australia; ^2^Language Research Laboratory, Department of Psychology, Cleveland State University, ClevelandOH, USA

**Keywords:** Stroop effect, reverse Stroop effect, strength of association, translation, reading, color

## Abstract

In a reverse Stroop task, observers respond to the meaning of a color word irrespective of the color in which the word is printed—for example, the word *red* may be printed in the congruent color (red), an incongruent color (e.g., blue), or a neutral color (e.g., white). Although reading of color words in this task is often thought to be neither facilitated by congruent print colors nor interfered with incongruent print colors, this interference has been detected by using a response method that does not give any bias in favor of processing of word meanings or processing of print colors. On the other hand, evidence for the presence of facilitation in this task has been scarce, even though this facilitation is theoretically possible. By modifying the task such that participants respond to a stimulus color word by pointing to a corresponding response word on a computer screen with a mouse, the present study investigated the possibility that not only interference but also facilitation would take place in a reverse Stroop task. Importantly, in this study, participants’ responses were dynamically tracked by recording the entire trajectories of the mouse. Arguably, this method provided richer information about participants’ performance than traditional measures such as reaction time and accuracy, allowing for more detailed (and thus potentially more sensitive) investigation of facilitation and interference in the reverse Stroop task. These trajectories showed that the mouse’s approach toward correct response words was significantly delayed by incongruent print colors but not affected by congruent print colors, demonstrating that only interference, not facilitation, was present in the current task. Implications of these findings are discussed within a theoretical framework in which the strength of association between a task and its response method plays a critical role in determining how word meanings and print colors interact in reverse Stroop tasks.

## Introduction

When observers are presented with a color word and asked to indicate in what color the word is written (e.g., if the word *red* is printed in blue ink, the correct answer is blue, not red), the incongruence of the meaning of the word (red) and the print color (blue) adversely affects the observers’ performance (Stroop, 1935). This so-called Stroop effect is one of the most extensively studied phenomena in the history of experimental psychology (see MacLeod, 1991, for a review). On the other hand, the reverse of the above task (i.e., observers respond to the same kind of stimuli by reading color words, not by identifying their print colors) has received much less attention in the literature. One possible reason for the lack of interest in this word-reading task is that incongruent print colors typically do not interfere with observers’ reading of the color words. That is, the observers are able to read aloud the color words equally quickly regardless of the colors those words are printed in (e.g., Stroop, 1935; Simon and Sudalaimuthu, 1979; Dunbar and MacLeod, 1984; Virzi and Egeth, 1985; MacLeod, 1991; Blais and Besner, 2006). The present study was designed to revisit the word-reading Stroop task (i.e., the reverse Stroop task) and examine the potential for an interaction between the word meanings and print colors in this task. Specifically, we examined whether incongruent print colors would interfere with reading of color words, as well as whether congruent print colors (e.g., *red* is printed in red ink) would facilitate reading of color words.

The absence of reverse Stroop effects (i.e., print colors causing no interference with reading of color words), as well as the presence of Stroop effects, are often explained by positing that interference occurs in these tasks only when a correct response entails translation of task-relevant information from one format of representation into another (translation hypothesis; Virzi and Egeth, 1985; Glaser and Glaser, 1989; Sugg and McDonald, 1994). For example, in a standard version of the Stroop task in which participants vocally name print colors, visual information of the print colors needs to be translated into verbal codes when participants make vocal responses. This translation gives rise to interference of the meanings of color words with the print colors. On the other hand, in a typical reverse Stroop task in which participants read aloud color words, no such translation between visual and verbal codes is involved because the color words are already represented in the verbal codes that are used for responses (i.e., in this case, only translation from orthographic to phonological codes is required). Thus, print colors and the meanings of the color words do not interact, and no reverse Stroop effects would emerge.

Following the same logic, the translation hypothesis can be extended to state that no interference of print colors with word meanings should be present in reverse Stroop tasks as long as correct responses can be determined by using verbal information (of the meanings of color words) alone; that is, vocal responses (i.e., reading aloud stimulus color words) are not required for reverse Stroop effects to be absent in these tasks. For example, when the response method is changed to pressing buttons that are labeled with the same color words as the stimulus words, the print colors of the stimulus words should continue to have no influence on participants’ performance. However, this is not always the case. Blais and Besner (2006) used exactly this version of the reverse Stroop task and found that stimulus words that were printed in incongruent colors elicited slower responses than stimulus words that were printed in congruent colors. Subsequently, Blais and Besner (2007) modified the task in such a way that participants responded to a stimulus color word by moving a mouse cursor to a corresponding response color word displayed on a computer screen, and demonstrated that this method also resulted in delayed responses to stimulus color words printed in incongruent colors (not only compared to responses to stimulus color words printed in congruent colors, but also relative to responses to stimulus color words printed in a neutral white color). Together, these results demonstrated the reverse Stroop effects when tasks did not require translation of the verbal information of stimulus color words into another representational format, suggesting that the translation hypothesis does not fully explain how word meanings and print colors would (or would not) interact in reverse Stroop tasks.

To account for reverse Stroop effects in the absence of translation, Blais and Besner (2006, 2007) proposed an alternative hypothesis in which the strength of association between a task and its response method plays an important role. This hypothesis focuses on the fact that participants have a natural propensity to be more skilled at carrying out certain processes than others, and posits that more skilled processes have impact on less skilled processes. For example, when word reading and print color naming are combined with vocal responses (i.e., typical Stroop and reverse Stroop tasks), participants are likely to be more practiced at reading words than naming colors through their daily activities. As a result, in the Stroop task the more skilled word-reading process interferes with the less skilled color-naming process (producing Stroop effects), but in the reverse Stroop task the color-naming process is much less likely to exert a detectable influence on the word-reading process (causing the lack of reverse Stroop effects). Importantly, central to this hypothesis, the degree to which participants are skilled at different processes depends on what response methods are used. When the same word-reading and color-naming processes are paired with manual responses such as pressing buttons labeled with color words (Blais and Besner, 2006) and pointing to color words with a mouse (Blais and Besner, 2007), the advantage of the word-reading process over the color-naming process diminishes because it is hard to assume that participants are more practiced at reading a word and making an associated manual response than identifying a print color and making the same manual response. Thus, in these manual versions of the reverse Stroop task, there can be some room for the color-naming process to interfere with the word-reading process (yielding reverse Stroop effects).

Notably, according to the strength-of-association hypothesis summarized above, interaction between word reading and color naming can go either way when participants are equally (un)skilled at these processes—that is, the hypothesis does not specify that the interaction has to manifest itself in the form of interference between the processes; instead, it is theoretically possible that the two processes interact by facilitating each other, as long as tasks use unbiased response methods with which one process is not more practiced than the other. Nevertheless, to our knowledge, there is only limited evidence for this facilitation in reverse Stroop tasks. The scarcity of the evidence is partly due to the simple fact that the facilitation effect has yet to be explicitly investigated. Previous studies that measured reverse Stroop performance by using unbiased response methods (Pritchatt, 1968; Durgin, 2000; Ruff et al., 2001; Blais and Besner, 2006) tended to focus exclusively on interference effects by including only incongruent conditions (in which color words were printed in incongruent colors) and neutral conditions (in which color words were printed in a neutral color such as white). Alternatively, when previous studies did include congruent conditions (in which color words were printed in congruent colors), those studies omitted neutral conditions and directly compared congruent and incongruent conditions (Simon and Sudalaimuthu, 1979; Virzi and Egeth, 1985; Simon and Berbaum, 1990; Melara and Mounts, 1993; Lu and Proctor, 2001; West, 2004; Blais and Besner, 2006). Because these direct comparisons could only identify a mixture of facilitation and interference effects, and furthermore, because any difference between congruent and incongruent conditions was typically interpreted as the result of interference between word meanings and print colors, it has not been specifically explored whether congruent print colors can facilitate reading of color words when unbiased response methods are employed.

Moreover, a few studies that were appropriately designed for the possibility of detecting facilitation in reverse Stroop tasks (i.e., studies that compared congruent and neutral conditions by using unbiased response methods) have not led to an unequivocal conclusion. As described previously, Blais and Besner (2007) used a mouse-pointing version of the reverse Stroop task and only found interference of incongruent print colors with reading of color words; responses to stimulus words printed in congruent colors were no faster than responses to stimulus words printed in a neutral color. Nevertheless, when Blais and Besner (2007) simulated their experiment within the framework of the strength-of-association hypothesis [by using a computational model of Stroop performance formulated by Cohen et al. (1990)], the simulation not only replicated interference of the empirically observed magnitude, but also yielded small yet reliable facilitation in reading of congruently colored stimulus words relative to reading of neutrally colored stimulus words. This simulation suggests that the facilitation effect could have been observed in the experiment, making it unclear why facilitation was not detected in Blais and Besner’s (2007) reverse Stroop task.

Another notable instance can be found in the study conducted by Sugg and McDonald (1994). In their experiments, stimulus color words printed in white were presented inside of rectangles whose border colors were either congruent, incongruent, or neutral to the meanings of the stimulus words. When participants responded to the word meanings by manually pressing correspondingly colored buttons, facilitation was found between congruent and neutral conditions (especially when the stimulus rectangles appeared earlier than the stimulus words). However, the facilitation effect disappeared when the colored response buttons were replaced with verbally labeled response buttons. According to the strength-of-association hypothesis, this change in the response methods should not have caused such a striking difference in participants’ performance because there is no clear reason to assume that the participants could have had differential skill levels in pressing colored buttons and verbally labeled buttons in response to the same stimuli. As such, findings from the Sugg and McDonald study are merely suggestive of facilitation in reverse Stroop tasks, calling for further investigation as to whether reading of color words can truly be facilitated by the presence of congruent colors.

In sum, none of the previous studies discussed above found distinct evidence for facilitation in reverse Stroop tasks. This result might well be interpreted as suggesting the true absence of facilitation in reverse Stroop tasks. However, before coming to this conclusion, one factor should receive careful consideration: although the previous studies used a variety of response methods (such as vocal response, button press, and pointing by a mouse), they all assessed participants’ performance by measuring how quickly and accurately the participants made responses to various types of stimulus color words (i.e., reaction time and accuracy). It should be noted that these are end-point measures—effects of all processes that take place in the course of a trial accumulate to one point of measurement, which is taken at the end of the trial. Therefore, it is possible that the reverse Stroop tasks used in the previous studies did indeed involve facilitation, but the effects of facilitation were masked by effects of other processes that were of greater influence on reaction time and accuracy. This is a real possibility, given that the facilitation effects, if they exist, would most likely be small (otherwise, the previous studies would have revealed them in an unequivocal manner). From this standpoint, it can be argued that a potentially more informative approach to examining the facilitation effects is to monitor participants’ performance throughout a trial as the participants carry out the mental processes associated with forming and executing a response.

To this end, the present study employed a new version of the reverse Stroop task in which participants’ responses were dynamically captured as they unfolded. The participants responded to the meanings of stimulus color words by moving a mouse cursor to corresponding response color words on a computer screen, and while the response was being carried out, the entire trajectory of the mouse cursor was recorded. If congruently colored stimulus words elicited facilitated responses, the mouse trajectories would exhibit more efficient movements toward correct response words compared to mouse trajectories that were formed in response to neutrally colored stimulus words. Similarly, if incongruently colored stimulus words caused interference between word meanings and print colors, such interference would be characterized by less efficient trajectories toward correct response words. Importantly, in this approach, the efficiency of the mouse trajectories can be quantified not only by the time it takes for a mouse cursor to reach correct response words (i.e., reaction time) but also by geometric and kinematic properties of the trajectories. For example, when responses are made more efficiently, the cursor would start approaching correct response words more rapidly. In this case, changes in moment-by-moment positions of the cursor would reveal facilitation of the responses, even though these trajectories might remain statistically equivalent to non-facilitated trajectories in terms of overall reaction times.

It is also important to note that the current mouse-pointing response method should be neutral to meanings and print colors of stimulus color words. That is, it is unreasonable to assume that participants’ skill levels in moving a mouse would vary depending on which of the two stimulus dimensions was processed. Furthermore, the use of response color words instead of colors themselves (e.g., colored shapes) enabled participants to select correct responses by using verbal information alone. This manipulation made translation between verbal and visual codes unnecessary, allowing the present study to focus on the strength-of-association hypothesis. Following this hypothesis, it was predicted that the meanings of the stimulus words and their print colors would interact and produce not only interference in an incongruent condition, but also possibly facilitation in a congruent condition. These facilitation and interference effects were examined by comparing the congruent and incongruent conditions against a neutral condition. The effects were quantified by mouse trajectories, in addition to traditional measures of reaction time and accuracy.

## Materials and Methods

The experiment reported below was approved by the Institutional Review Board at Cleveland State University. All data were collected in the Language Research Laboratory at Cleveland State University.

### Participants

Twenty-two native English speakers (4 men and 18 women; mean age = 20.8 years old) who reported no impairments of vision, hearing, speech, or reading were recruited from the Cleveland State University community. These participants exhibited clear right-handedness in a modified version of the Edinburgh inventory^1^ (Oldfield, 1971): *M* = 80.91, *SD* = 17.84 (scores range from -100 to 100 in this inventory, with larger scores signifying greater degrees of right laterality). All participants gave written informed consent to participate in this study and received research participation credit in psychology courses.

### Stimuli and Design

One of four color words (*blue*, *green*, *red*, and *yellow*) was presented as a stimulus in upright uppercase letters against a black background. The stimulus words were written in 44-point Arial font in the middle of a 17-inch screen. Participants were seated approximately 60-cm away from the screen. At this viewing distance, the stimulus words were approximately 1.0° tall and 2.9–3.8° wide in visual angles. There were 32 trials in each of the three conditions (congruent, incongruent, and neutral). The four stimulus words were used eight times apiece in each condition. In the incongruent condition, each stimulus word appeared in all three incongruent colors across trials (i.e., three colors out of blue, green, red, and yellow, except the one congruent to the meaning of the stimulus word). In the neutral condition, all stimulus words were written in white.

**FIGURE 1:**
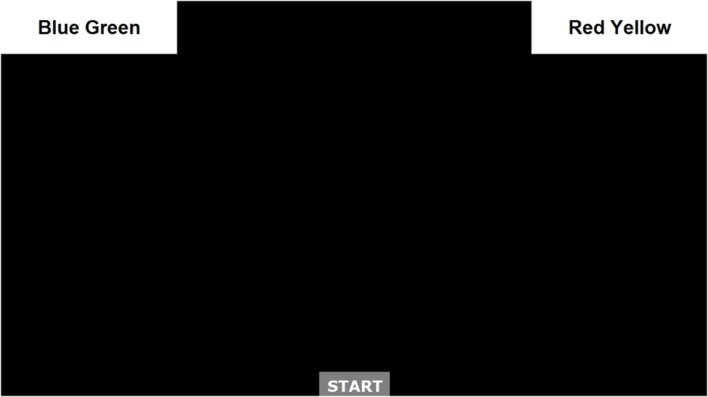
**Graphical presentation of the task.** Participants clicked the gray START button to begin a trial. A stimulus color word (not shown in the figure) appeared immediately at the center of the screen and remained visible until participants made a response. In the two white rectangles at the upper corners of the screen, response color words were presented in pairs. To make a response, participants moved a mouse and clicked one of the white rectangles.

In each trial, the same four color words were also presented at two upper corners of the screen, to which participants moved a mouse cursor to make a response (**Figure 1**). These response color words were written in black within white rectangles using 44-point Arial font (with only the initial letter of each word capitalized). When viewed at 60 cm, the white rectangle was approximately 2.8° tall and 9° wide. Each white rectangle contained two response color words. The pairing of the response color words was determined so that only one word of a pair always gave a correct response. For example, the blue-green pair was not used when the stimulus word *blue* was printed in green. This layout of the response words might have increased the complexity of response selection, but previous studies used similar mappings of response options and successfully demonstrated Stroop and reverse Stroop effects (e.g., De Houwer, 2003; Schmidt and Cheesman, 2005; Blais and Besner, 2006; Incera and McLennan, 2016).

Out of 24 possible ways of arranging the four response color words, the following four configurations were used: Blue Green and Red Yellow (as shown in **Figure 1**); Green Red and Yellow Blue; Red Yellow and Blue Green; and Yellow Blue and Green Red. These configurations were used in this order in four blocks of the experiment. At the beginning of a block (before starting trials), participants were allowed to familiarize themselves with the positions of the response words on the screen. In each block, eight congruent trials, eight incongruent trials, and eight neutral trials were randomly presented. Within each trial type, the four stimulus color words were shown in a random order. The print colors of incongruent stimuli were also selected randomly for each trial with the constraints stated previously—that is, (a) a response color word corresponding to the print color was not paired with a response color word corresponding to the meaning of a stimulus color word; and (b) all blocks combined, each stimulus word was printed in all three incongruent colors across trials.

Prior to performing the four blocks of experimental trials, participants carried out four practice trials in which they learned about the general task procedure. In these trials, stimuli consisted of strings of symbols (^∗∗∗^, +++, ###, and %%%) written in white. The same strings also appeared (in black) as response options as in the experimental trials. Each of the stimulus strings was used once and presented in a random order.

### Procedure

To begin a trial, participants clicked the START button on the screen (see **Figure 1**). This click initialized the position of a mouse cursor at the bottom center of the screen. A stimulus color word was presented without any delay and remained on the screen until participants selected a response color word. To make this selection, participants were allowed to click anywhere in the white rectangle that contained the response word (i.e., it was not necessary to click on the response word itself), and participants were instructed to do so as quickly and accurately as possible. During the trial, positions of the mouse cursor were recorded every 13–16 ms by using the MouseTracker program^2^ (Freeman and Ambady, 2010). Reaction time was also measured as the time elapsed between the first mouse click on the START button and the second mouse click on one of the white rectangles.

Participants were instructed to start moving the mouse as soon as they received a stimulus color word. When no mouse movement was detected within 500 ms of the beginning of a trial, a message that asked participants to begin moving the mouse earlier was displayed on the screen after participants clicked one of the response options. Otherwise, this click immediately triggered the next trial by displaying the START button again. This instruction was to prevent participants from mentally selecting a response first and then making a ballistic movement of the mouse—responses made in such a manner would be problematic to the purpose of the present experiment, which was to dynamically capture participants’ responses as they were formed and executed. Other than the possible warning message, no performance feedback was given to participants during the experiment.

## Results

### Data Screening

Out of the total of 2,112 trials (i.e., 96 trials per participant), 34 trials were excluded from the final analyses. First, 21 trials in which participants made incorrect responses were discarded. Second, out of the remaining trials, six trials were removed because initiation times (i.e., the time elapsed between the beginning of a trial and the first detected mouse movement) were greater than 500 ms, suggesting that mouse trajectories in these trials did not fully capture participants’ responses. Third, seven more trials were removed because of erratic (and thus uninterpretable) mouse trajectories—these trajectories moved back and forth, forming a loop by crossing themselves. The removed trials constituted a very small portion of the correctly performed trials (0.62%) and were distributed evenly across congruent, incongruent, and neutral conditions (for details, see **Supplementary Table S1**).

### Initiation Time

**FIGURE 2:**
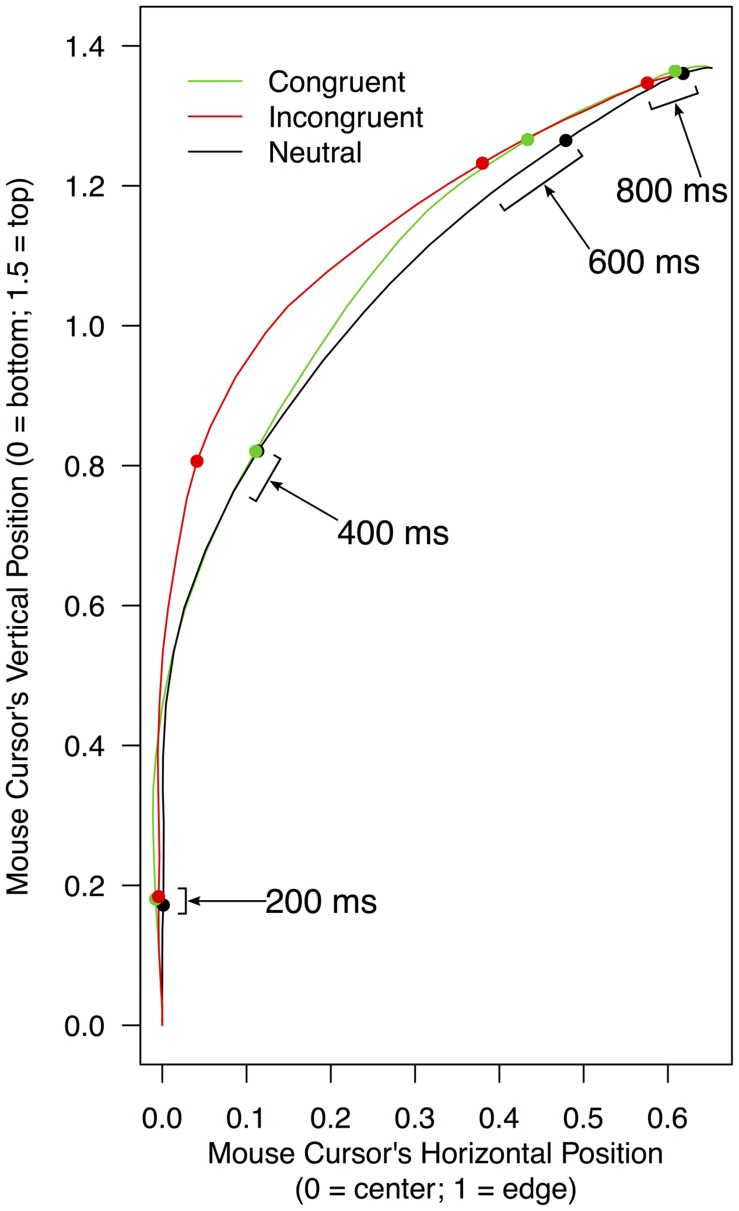
**Mean trajectories of a mouse cursor on a screen as a function of experimental conditions.** To compute these mean trajectories, leftward trajectories were bilaterally flipped and pooled with rightward trajectories (for details, see section “Mouse Trajectory” under “RESULTS” of the text). Filled dots indicate mean mouse positions in each condition for every 200 ms since the beginning of a trial.

**FIGURE 3:**
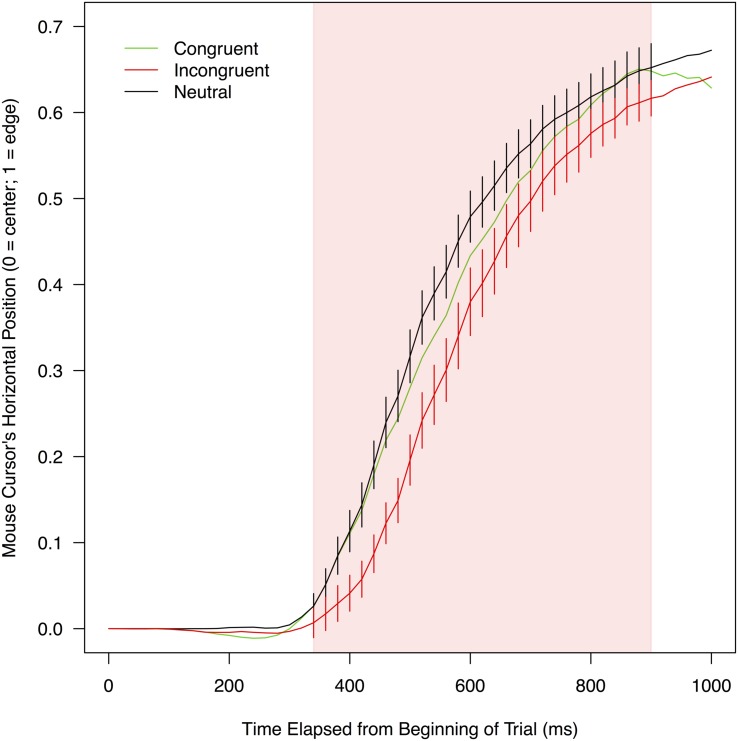
**Mean horizontal positions of a mouse cursor on a screen as a function of experimental conditions and the time elapsed from the beginning of a trial.** To compute these means, leftward trajectories were bilaterally flipped and pooled with rightward trajectories (for details, see section “Mouse Trajectory” under “RESULTS” of the text). The red shade indicates the time range (320–900 ms) in which mouse movements toward correct responses were significantly delayed when stimulus color words were written in incongruent colors (compared to when the stimulus words were neutrally colored in white). Error bars shown in this range represent 95% confidence intervals.

**FIGURE 4:**
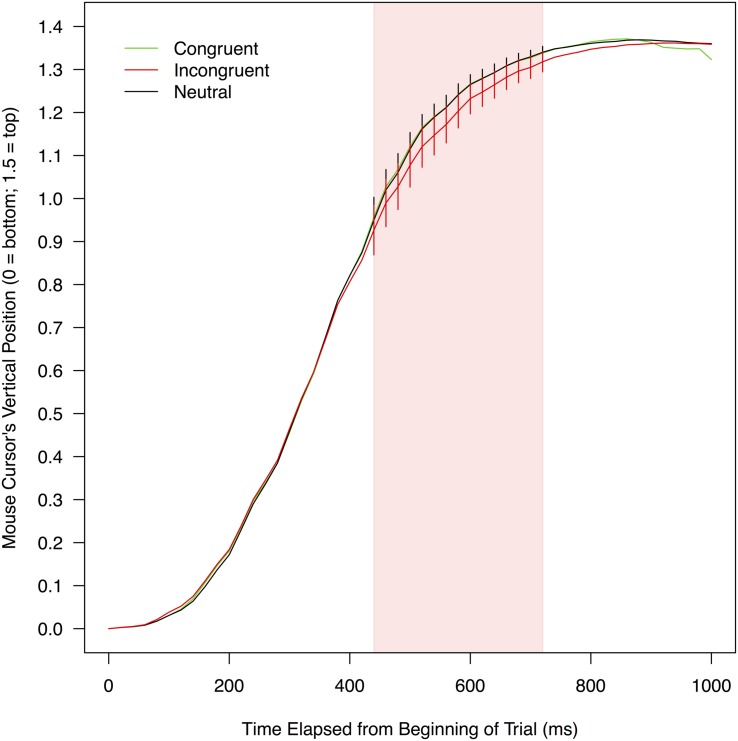
**Mean vertical positions of a mouse cursor on a screen as a function of experimental conditions and the time elapsed from the beginning of a trial.** To compute these means, leftward trajectories were bilaterally flipped and pooled with rightward trajectories (for details, see section “Mouse Trajectory” under “RESULTS” of the text). The red shade indicates the time range (440–740 ms) in which mouse movements toward correct responses were significantly delayed when stimulus color words were written in incongruent colors (compared to when the stimulus words were neutrally colored in white). Error bars shown in this range represent 95% confidence intervals.

Initiation times in each condition were as follows: *M* = 119.76 ms, *SD* = 61.05 ms (congruent); *M* = 117.40 ms, *SD* = 59.64 ms (incongruent); *M* = 118.88 ms, *SD* = 58.02 ms (neutral). To confirm that mouse movements began at equivalent times in all conditions, initiation times were subjected to a repeated measures analysis of variance with condition (congruent, incongruent, and neutral) as a sole factor. This analysis did not reveal a reliable effect, *F*(2,42) = 0.288, *p* = 0.751, 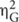 < 0.001 (for details about 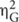, see Bakeman, 2005), suggesting that the three conditions did not differ in how soon participants started moving a mouse after beginning a trial. As can be seen in mouse trajectory data (details are shown below in section “Mouse Trajectory” under “RESULTS” and **Figures 2–4**), participants generally moved the mouse vertically along the central line of the screen during the initial period of responses (i.e., without moving toward either of the two pairs of response words).

### Reaction Time and Accuracy

**Table 1** shows mean reaction times and accuracy in each of the three conditions. Mean reaction times and accuracy were analyzed separately by planned contrasts in which two conditions of interest were directly compared: congruent versus neutral conditions and incongruent versus neutral conditions for examining possible facilitation and interference effects, respectively. Congruent and incongruent conditions were not directly compared because, as discussed previously, any difference revealed by this comparison cannot be attributed uniquely to either facilitation or interference effects.

**Table 1:** Mean reaction times and accuracy in three conditions of the experiment.

	Reaction time (ms)		Accuracy (%)	
		
	*M*	*SD*	*M*	*SD*
Congruent	984.06	134.35	99.29	1.34
Incongruent	1,046.73	160.51	98.30	2.68
Neutral	986.31	141.62	99.43	1.57


Reaction times in the congruent condition did not differ significantly from those in the neutral condition, *t*(21) = 0.204, *p* = 0.841, *d* = 0.016. On the other hand, reaction times in the incongruent condition were significantly longer than those in the neutral condition, *t*(21) = 4.430, *p* < 0.001, *d* = 0.399. Thus, these results showed the typical pattern of reaction time data in reverse Stroop tasks—that is, reading of color words was significantly slowed down by incongruent print colors, whereas the same reading process was not sped up by congruent print colors.

Accuracy data showed that participants performed the task with a high degree of accuracy in all conditions. There was no reliable difference between congruent and neutral conditions, *t*(21) = 0.326, *p* = 0.747, *d* = 0.097, or between incongruent and neutral conditions, *t*(21) = -1.702, *p* = 0.104, *d* = -0.518. Given the nature of the current task, such high overall accuracy (and thus the lack of differences between conditions due to ceiling effects) was expected (Blais and Besner, 2007).

### Mouse Trajectory

The horizontal mouse positions were expressed in numerical values that varied from -1 (the left edge of the screen) to 1 (the right edge of the screen), with 0 being the middle (the START button). Similarly, the vertical mouse positions ranged from 0 (the bottom of the screen) to 1.5 (the top of the screen). Given that participants were allowed to click anywhere within a white rectangle containing response words, it was expected that few trajectories would reach the edge of the screen. Expressed in the same numerical scales, the horizontal centers of the two white rectangles were at -0.75 (left) and 0.75 (right); and their vertical centers were at 1.4. Initially, trajectories that progressed toward the left rectangle and those that progressed toward the right rectangle were analyzed separately. However, the two groups of trajectories yielded the same patterns, and thus detailed analyses reported below were conducted by pooling all trajectories (i.e., the leftward trajectories were bilaterally flipped around the vertical central line of the screen). **Figure 2** shows mean mouse trajectories in each of the three conditions (congruent, incongruent, and neutral) collapsed over 22 participants. These trajectories suggest that (a) the mouse cursor approached a correct response in a similar way in the congruent and neutral conditions; and (b) mouse movements toward a correct response were delayed in the incongruent condition compared to the neutral condition. As shown below, formal analyses confirmed these observations.

#### Horizontal Mouse Position

Analysis of mouse trajectories was primarily focused on horizontal mouse positions because the two response options were separated horizontally, but they were in the same vertical position of the screen. Mean trajectories in each of the three conditions were computed for each participant, and like in the analyses of reaction times and accuracy, these mean trajectories were compared between congruent and neutral conditions (for facilitation effects) as well as between incongruent and neutral conditions (for interference effects). The trajectories were divided into a series of 20-ms time bins, and mean horizontal mouse positions within each time bin were contrasted between two relevant conditions by a paired *t*-test. The trajectories differed in their total durations, but no participant finished moving the mouse in all trials within 1,000 ms of the beginning of a trial. Thus, the 50 bins from the initial second of the trials were used for the trajectory analyses. Given that mean reaction times in the three conditions were 984–1,047 ms, these 50 bins covered most portions of the trajectories. **Figure 3** displays mean horizontal mouse positions in the 50 bins collapsed over participants as a function of the conditions.

To determine whether trajectories in two relevant conditions were reliably different, a statistical threshold was established on the basis of the number of *t*-tests that showed significant differences (each at α = 0.05) between the conditions in contiguous time bins. This threshold was used in order to keep the overall Type-I error rate lower than 0.05, despite the fact that data were analyzed with multiple *t*-tests. To set the threshold, a simulation-based technique developed by Dale et al. (2007) was adopted. In this technique, for each time bin and for each condition, 22 data points (each of which represented the mean horizontal mouse position of one simulated participant) were randomly sampled from a normal distribution whose mean and standard deviation were identical to those of the actual data in the corresponding time bin and condition of the present experiment. By sampling from the distributions of the actual data, the simulated data reflected the fact that horizontal mouse positions in each time bin were not independent of one another (i.e., time bins that were close to each other tended to have similar horizontal mouse positions). The simulated data were analyzed by *t*-tests in the same manner as the experimental data. This procedure was repeated 10,000 times in total, and the threshold was defined as the smallest number of consecutively significant *t*-tests that appeared less than 500 times in the 10,000 simulated experiments (i.e., 500/10,000 = 0.05). In other words, an observed divergence between trajectories in the actual data was not considered significant unless (a) a *t*-test indicated statistically reliable (*p* < 0.05) separation between the trajectories in a given time bin and (b) time bins in which *t*-tests were significant formed an uninterrupted sequence that was longer than the defined threshold. When the sequence of significant *t*-tests in the experimental data was longer than this threshold, the probability of its happening by chance would be less than 0.05. Results of this simulation are shown in **Supplementary Table S2**.

Regarding possible facilitation effects, horizontal mouse positions were not significantly closer to a correct response word in the congruent condition than in the neutral condition in any of the 50 time bins, *t*s(21) < 0.614, *p*s > 0.273, *d*s < 0.401. Thus, it was not necessary to conduct the above-mentioned simulation. This result indicates that congruent print colors did not exert any reliable facilitation effects on the horizontal mouse positions.

As for possible interference effects, horizontal mouse positions were significantly farther away from a correct response word in the incongruent condition than in the neutral condition in 29 contiguous time bins between 320 and 900 ms, *t*s(21) < -1.770, *p*s < 0.046, *d*s > 0.307. In this time period (indicated by the red shade in **Figure 3**), mean horizontal mouse positions ranged from 0.01 to 0.62 in the incongruent condition (*M* = 0.35, *SD* = 0.22), and from 0.03 to 0.65 in the neutral condition (*M* = 0.43, *SD* = 0.20). The simulation set the threshold for this comparison at 14 consecutively significant *t*-tests (see **Supplementary Table S2** for details). Therefore, in these 29 time bins, mouse movements to the correct response option were significantly less efficient in the incongruent condition than in the neutral condition, showing that there was reliable interference between the meanings of stimulus color words and their print colors.

#### Vertical Mouse Position

**Figure 4** shows mean vertical positions of a mouse collapsed over participants as a function of the three conditions. The vertical mouse positions were analyzed in the same manner as the horizontal mouse positions. There were no significant differences between the congruent and neutral conditions in any of the 50 time bins, *t*s(21) < 1.343, *p*s > 0.096, *d*s < 0.212, exhibiting no reliable signs of facilitation. The vertical mouse positions in the incongruent condition were significantly farther away from a correct response option than those in the neutral condition in 15 consecutive bins between 440 and 740 ms, *t*s(21) < -1.821, *p*s < 0.042, *d*s > 0.110. In this time period (indicated by the red shade in **Figure 4**), mean vertical mouse positions ranged from 0.93 to 1.32 in the incongruent condition (*M* = 1.17, *SD* = 0.12), and from 0.95 to 1.34 in the neutral condition (*M* = 1.21, *SD* = 0.12). According to the simulation, the threshold for this comparison was the sequence of four significant *t*-tests (see **Supplementary Table S2** for details). Thus, it was concluded that mouse movements toward correct responses were reliably delayed in the incongruent condition relative to the neutral condition, providing evidence that the print colors of stimulus words interfered with the word meanings.

#### Velocity and Acceleration of Mouse Movement

The magnitudes of velocity and acceleration of a mouse along its trajectory were also analyzed in the same manner as the horizontal and vertical mouse positions. These analyses did not reveal any reliable facilitation and interference. Some *t*-tests showed significant differences between two conditions of interest in given time bins, but no sequence of significant *t*-tests was deemed significant when evaluated by the same simulation-based method.

## Discussion

The present study was designed to investigate whether meanings of color words and their print colors on a screen would interact with each other in a reverse Stroop task. According to the strength-of-association hypothesis (Blais and Besner, 2006, 2007), processing of the two dimensions of the stimulus color words can be both facilitated and interfered by one another as long as participants’ skills in responding to each of these dimensions are equated by using an unbiased response method. The current experiment employed one such method (i.e., having participants move a mouse cursor to an appropriate response color word on the screen), and thus it was predicted that congruently colored stimulus words could be read more efficiently than neutrally colored stimulus words (facilitation), and that incongruently colored stimulus words would be read less efficiently than neutrally colored stimulus words (interference). To test these predictions, participants’ performance was evaluated by analyzing trajectories of the mouse (in addition to reaction time and accuracy). Mouse trajectory data showed that (a) the mouse approached a correct response word at an equivalent rate in the congruent and neutral conditions and (b) mouse movement toward a correct response word was significantly delayed in the incongruent condition relative to the neutral condition, revealing no facilitation and reliable interference effects, respectively. Accuracy data did not show any effects, but reaction time data were consistent with the mouse trajectory data in that reaction times were statistically indistinguishable between the congruent and neutral conditions (i.e., no facilitation), whereas reaction times were significantly longer in the incongruent condition than in the neutral condition (interference). These results and their theoretical implications are discussed in detail below.

### Reaction Time and Accuracy

Accuracy data were not clear about whether reading of stimulus color words was reliably affected by congruent and incongruent print colors. Participants made few errors in the current task irrespective of conditions, making the accuracy data insensitive to the manipulation of the print colors. This result was expected (and consistent with previous findings; e.g., Blais and Besner, 2007), given that the task was not difficult—both stimulus and response words were presented clearly and participants performed the task under little or no time pressure (i.e., they were instructed to make an accurate response as quickly as possible, but there was no time limit on each trial). The reaction time data showed that there was no reliable difference between congruent and neutral conditions (suggesting the absence of facilitation), and that participants’ responses were significantly slower in the incongruent condition than in the neutral condition (suggesting the presence of interference). These results were also consonant with previous results from reverse Stroop tasks in which interference effects were reliably present but facilitation effects were found only sporadically at best (Sugg and McDonald, 1994; Blais and Besner, 2007). In sum, the present results from these traditional measures accord well with previous findings in the literature, suggesting that despite the introduction of the novel mouse-tracking paradigm, the current experiment faithfully implemented the reverse Stroop task without altering its underlying mental processes.

### Mouse Trajectory

As summarized above, mouse trajectory data showed that there was interference but not facilitation between meanings of stimulus color words and their print colors in the current version of the reverse Stroop task. One notable aspect of the data is that facilitation effects were virtually non-existent in any of the analyses conducted in the present study. As shown in **Figure 2**, trajectories in the congruent and neutral conditions resembled each other in terms of both their overall shapes and how quickly participants moved a mouse along them. Thorough statistical analyses were conducted not only on the horizontal mouse positions (i.e., the dimension that is supposedly most sensitive to the effects of facilitation and interference in the current paradigm) but also on the vertical mouse positions as well as the magnitudes of velocity and acceleration of the mouse, and none of these analyses yielded evidence for the presence of facilitation. Importantly, in these mouse trajectory analyses the entire time course of a response was examined, not just the total time taken to make the response (i.e., reaction time). These converging (null) findings from the fine-grained analyses strongly suggest that as compared to the neutral white color, the congruent colors did not exert any additional effects on response processes in the present experiment.

By contrast, the current results revealed clear evidence for interference between word meanings and print colors. Mouse movements toward correct responses were significantly delayed in the incongruent condition (relative to the neutral condition) not only along the horizontal dimension (**Figure 3**) but also along the vertical dimension (**Figure 4**). The effect of interference was particularly evident in the horizontal dimension, in which the effect emerged early (at 320 ms since the beginning of a trial) and sustained through the trial (up to 900 ms; the mean length of incongruent trials was 1,046.73 ms). These results showed that the incongruent print colors robustly interfered with the reading of stimulus color words in this experiment.

The early appearance of interference effects in the horizontal mouse positions helps exclude one explanation for the lack of facilitation in the present study. In the Stroop literature, several studies have proposed that effects of one stimulus dimension on the other (i.e., word meanings and print colors) surface only when the relevant dimension is fully processed during a trial (e.g., Dyer, 1973; Morton and Chambers, 1973; Palef and Olson, 1975; Posner, 1978). Because naming a color usually takes longer than reading a word (Cattell, 1886; Ferrand, 1999), this proposal would lead to the prediction that trials need to take long enough for print colors to exert any observable effects on participants’ performance in Stroop and reverse Stroop tasks. On the basis of this idea, one might argue that trials in the current experiment were too short to let facilitation effects emerge. However, interference effects appeared as early as 320 ms after the beginning of a trial in this experiment, suggesting that by this time the print colors were processed to the degree that was sufficient to affect the processing of the word meanings. Nevertheless, throughout the initial 1,000-ms period of the trials, the interaction between the word meanings and print colors was observed only in the form of interference. Furthermore, it should also be noted that the duration of the current reverse Stroop task was longer, not shorter, than other versions of the task used in previous studies—the mean overall reaction time in the present study was 1,005.70 ms; on the other hand, reaction times in the previous studies of reverse Stroop tasks approximately ranged between 550 and 800 ms (Sugg and McDonald, 1994; Blais and Besner, 2007). Therefore, even if the temporal extent of trials were a factor, the present study would have been more sensitive to possible facilitation effects than the previous studies. Taken together, it is unlikely that the current findings could be explained simply by how long the reverse Stroop trials lasted in the experiment.

### Theoretical Implications of the Present Findings

Major findings from the present study are, as discussed in the previous sections, that (a) facilitation was not observed in the mouse-tracking version of the reverse Stroop task; and (b) interference did appear robustly in the same task. These findings have important implications for the two hypotheses of reverse Stroop performance discussed in this article—the strength-of-association hypothesis and the translation hypothesis. Regarding the strength-of-association hypothesis, the fact that interference was found in the current experiment is consonant with this hypothesis. According to this hypothesis, meanings of stimulus color words and their print colors should show evidence of interaction in reverse Stroop tasks as long as response methods are not biased toward responding to the meanings or responding to the print colors. Consistent with this prediction, reading of the stimulus words was interfered by the presence of incongruent print colors when participants made responses by moving a mouse cursor to response color words (i.e., a response method that was presumably neutral toward the two dimensions of the stimuli). This result adds to the body of new evidence for the strength-of-association hypothesis, lending additional support to the notion that the reverse Stroop interference is present even when a task does not entail translation of mental representations from one format to another (Blais and Besner, 2006, 2007).

However, not all of the current findings were supportive of the strength-of-association hypothesis. Importantly, within the framework of this hypothesis, there is no a priori reason to assume that the interaction between word meanings and print colors should occur only in the form of interference. Therefore, the fact that no study to date (Sugg and McDonald, 1994; Blais and Besner, 2007; and the present study) has yielded unequivocal evidence of facilitation in reverse Stroop tasks could raise a question as to whether the hypothesis should be modified to explain this absence of facilitation.

Because the lack of facilitation in reverse Stroop tasks, if firmly demonstrated, would have significant theoretical implications, great caution must be used when making such a conclusion. The present study made the first attempt in this regard by (a) providing a clear operational definition to facilitation in reverse Stroop tasks (by defining it as the difference between congruent and neutral conditions, not as the difference between congruent and incongruent conditions); (b) selecting a response method that was not biased toward processing of either word meanings or print colors; (c) designing the task so that it would not entail translation of representational format between visual and verbal codes; and (d) monitoring reverse Stroop performance dynamically throughout a response time course and characterizing interference (and facilitation, if it had been present) with multiple measures of mouse movement (in addition to response time and accuracy). The absence of facilitation as a result of this detailed investigation increases the likelihood that congruent print colors actually have no facilitatory effect on reading of color words. Nevertheless, further research would be needed before reaching a definitive conclusion. For example, in the present experiment, the three trial types (congruent, incongruent, and neutral) were randomly presented. This experimental design was justified because it effectively controlled for general practice effects (particularly important in the current experiment due to its novel response method), and followed a standard way of examining (reverse) Stroop effects by equating the trial types as to possible carry-over effects of previous trials on subsequent trials (MacLeod, 1991). However, it has been suggested that this design could degrade participants’ performance overall by increasing the cost of task switching between different types of trials (Wylie and Allport, 2000; Ward et al., 2001), which in turn could have had the effect of masking any subtle enhancement of performance (i.e., facilitation) in the present study. It would be informative to replicate the current experiment by manipulating the order of the trial types (e.g., by blocking and counterbalancing them).

Although the current findings are not completely decisive as to whether facilitation exists in reverse Stroop tasks, the results indicate that any effects of facilitation, if present, would be of significantly smaller magnitude than those of interference. This difference between facilitation and interference may be reflective of the same underlying mechanism that is activated to different degrees (Logan, 1980; Phaf et al., 1990). Alternatively, perhaps this difference is the result of having two qualitatively different underlying mechanisms (MacLeod and MacDonald, 2000). If so, the appearance of incongruent information might require a change in the course of a response, which would entail a great deal of mental effort (resulting in large interference); on the other hand, the presence of congruent information only aids in the execution of an existing response plan, providing relatively minor benefits (resulting in little or no facilitation). In other words, it would not be appropriate to conceptualize facilitation in (reverse) Stoop tasks as a simple parallel to interference. Future studies should compare the underlying dynamics of these two processes in order to be able to distinguish between these theoretical alternatives.

As for the translation hypothesis, the clear presence of interference in the present experiment poses a major challenge to the hypothesis. This hypothesis characterizes Stroop effects as the result of translating representation of task-relevant information (i.e., print colors of stimulus color words) from a visual code into verbal codes (Virzi and Egeth, 1985; Glaser and Glaser, 1989; Sugg and McDonald, 1994). The translation hypothesis predicts the absence of reverse Stroop effects because in typical reverse Stroop tasks no such translation of representational codes is necessary—in these tasks, participants are to read the stimulus color words, and therefore both stimulus encoding and response are carried out by using verbal codes. The need for the translation between visual and verbal codes was also excluded from the present experiment by having participants select color words displayed on a screen. Nevertheless, reading of the stimulus words was reliably affected by the incongruent print colors. Clearly, this finding does not accord well with the translation hypothesis.

To be fair, however, it should be pointed out that the translation between visual and verbal codes was not completely precluded from the current task; that is, the fact that this translation was not required does not necessarily indicate that it did not take place. Thus, there is still room for argument that some unknown factors in the present experiment made it possible for the translation to cause interference effects, even though such effects are usually not observable in typical reverse Stroop tasks. In the future, it may be worth combining the current paradigm with a technique through which the translation is explicitly suppressed. Such an approach would permit a more definitive conclusion as to the translation’s role (or lack thereof) in reverse Stroop tasks.

### Summary

In order to investigate the possibility that both facilitation and interference occur in a reverse Stroop task, the present study employed a mouse-tracking technique (Freeman and Ambady, 2010) to dynamically monitor participants’ responses as they were formed and executed. By examining the entire course of a response, this method was intended to enable a new approach toward exploring reverse Stroop effects in unprecedented detail. Results showed clear interference effects but no facilitation effects in any of the analyses. The presence of the robust interference provides support for the strength-of-association hypothesis (Blais and Besner, 2006, 2007), suggesting that word meanings and print colors do interact in reverse Stroop tasks when processing of one stimulus dimension is not given an advantage over processing of the other dimension through differential levels of prior practice (e.g., reading a word aloud versus vocally naming a color as in standard Stroop tasks—the former is typically more practiced than the latter). However, the absence of facilitation in the reverse Stroop tasks is incompatible with the current version of the hypothesis, calling for further investigation on this issue. The present study was among the first to attempt continuous monitoring of the temporal dynamics of reverse Stroop performance, and extension of this work has real potential to open up a new frontier of Stroop effect research.

## Author Contributions

All authors listed, have made substantial, direct and intellectual contribution to the work, and approved it for publication.

## Conflict of Interest Statement

The authors declare that the research was conducted in the absence of any commercial or financial relationships that could be construed as a potential conflict of interest.

## Footnotes

1 http://www.brainmapping.org/shared/Edinburgh.php

2 http://www.mousetracker.org/
