# Supplementary material for: A Reverse Stroop Task with Mouse Tracking
Source: Front Psychol. 2016 May 6;7:670. doi: 10.3389/fpsyg.2016.00670 (PMC4859192; doi:10.3389/fpsyg.2016.00670)
Supplement: Supplementary file 1 [file Table_1.PDF]

Supplementary Material

A Reverse Stroop Task with Mouse Tracking

Naohide Yamamoto[1,]*, Sara Incera[2], Conor T. M[c]Lennan[2]

[1]School of Psychology and Counselling and Institute of Health and Biomedical Innovation, Queensland University of Technology (QUT), Kelvin Grove, QLD, Australia
[2]Language Research Laboratory, Department of Psychology, Cleveland State University, Cleveland, OH, USA

*** Correspondence:** Naohide Yamamoto, School of Psychology and Counselling, Queensland University of Technology, Victoria Park Road, Kelvin Grove QLD 4059, Australia.
naohide.yamamoto@qut.edu.au

Supplementary Table S1. The numbers of discarded trials in each condition of the experiment. These trials were excluded from analysis for one of the following reasons: incorrect responses were made, mouse movements did not begin during the initial 500-ms period of a trial, and mouse trajectories were too erratic to be interpreted. For details, see section “Data Screening” of the main text.

	Congruent	Incongruent	Neutral	Total
**Incorrect response**	5	12	4	21
**Initiation time > 500 ms**	3	3	0	6
**Erratic trajectory**	3	3	1	7
**Total**	11	18	5	34
