# Supplementary material for: A Reverse Stroop Task with Mouse Tracking
Source: Front Psychol. 2016 May 6;7:670. doi: 10.3389/fpsyg.2016.00670 (PMC4859192; doi:10.3389/fpsyg.2016.00670)
Supplement: Supplementary file 2 [file Table_2.PDF]

frontiers

Supplementary Material

A Reverse Stroop Task with Mouse Tracking

Naohide Yamamoto[1,]*, Sara Incera[2], Conor T. McLennan[2]

[1]School of Psychology and Counselling and Institute of Health and Biomedical Innovation, Queensland University of Technology (QUT), Kelvin Grove, QLD, Australia
[2]Language Research Laboratory, Department of Psychology, Cleveland State University, Cleveland, OH, USA

*** Correspondence:** Naohide Yamamoto, School of Psychology and Counselling, Queensland University of Technology, Victoria Park Road, Kelvin Grove QLD 4059, Australia.
naohide.yamamoto@qut.edu.au

Supplementary Table S2. Sequences of significant *t*-tests that appeared in 10,000 simulated experiments. Frequencies of these consecutively significant *t*-tests were used for determining statistical thresholds in the present experiment. For details, see section "Mouse Trajectory" (under "RESULTS") of the main text.

Neutral vs. incongruent conditions (horizontal mouse positions)			Neutral vs. incongruent conditions (vertical mouse positions)		
**Number of consecutively significant *t*-tests**	**Frequency (out of 10,000)**	***p***	**Number of consecutively significant *t*-tests**	**Frequency (out of 10,000)**	***p***
13	864		2	5844	
14	464	< 0.05	3	921	
15	250		4	133	< 0.05
16	104		5	23	< 0.01
17	48	< 0.01	6	4	
